# Training, hypnosis, and drugs: artificial synaesthesia, or artificial paradises?

**DOI:** 10.3389/fpsyg.2013.00660

**Published:** 2013-10-14

**Authors:** Ophelia Deroy, Charles Spence

**Affiliations:** ^1^Centre for the Study of the Senses, School of Advanced Study, University of LondonLondon, UK; ^2^Department of Experimental Psychology, Crossmodal Research Laboratory, University of OxfordOxford, UK

**Keywords:** induced synaesthesia, synaesthesia, crossmodal correspondences, pseudo-synaesthesia, learning, genetic basis, drugs, hypnosis

## Abstract

The last few years have seen the publication of a number of studies by researchers claiming to have induced “synaesthesia,” “pseudo-synaesthesia,” or “synaesthesia-like” phenomena in non-synaesthetic participants. Although the intention of these studies has been to try and shed light on the way in which synaesthesia might have been acquired in developmental synaesthestes, we argue that they may only have documented a phenomenon that has elsewhere been accounted for in terms of the acquisition of sensory associations and is not evidently linked to synaesthesia. As synaesthesia remains largely defined in terms of the involuntary elicitation of conscious concurrents, we suggest that the theoretical rapprochement with synaesthesia (in any of its guises) is unnecessary, and potentially distracting. It might therefore, be less confusing if researchers were to avoid referring to synaesthesia when characterizing cases that lack robust evidence of a conscious manifestation. Even in the case of those other conditions for which conscious experiences are better evidenced, when training has been occurred during hypnotic suggestion, or when it has been combined with drugs, we argue that not every conscious manifestation should necessarily be counted as synaesthetic. Finally, we stress that cases of associative learning are unlikely to shed light on two highly specific characteristic of the majority of cases of developmental synaesthesia in terms of learning patterns: First, their resistance to change through exposure once the synaesthetic repertoire has been fixed; Second, the transfer of conditioned responses between concurrents and inducers after training. We conclude by questioning whether, in adulthood, it is ever possible to acquire the kind of synaesthesia that is typically documented in the developmental form of the condition. The available evidence instead seems to point to there being a critical period for the development of synaesthesia, probably only in those with a genetic predisposition to develop the condition.

## Introduction

In *Prometheus, Poem of Fire* (1910), Scriabin attempted to communicate to the audience the synaesthetic experience of colored musical keys that he claimed to have enjoyed for most of his life. A color organ was used to project colored lights during the concert (Peacock, 1985; see also Gawboy and Townsend, 2012, for a recent performance given at Yale). Critics left the first performance of Scriabin's work stressing that the lights had “no possible connection to the music,” and merely “served to divert the senses of the audience from a too concentrated attention on the music” (Clarence Lucas quoted in Hull, 1927, p. 227). Several other artists have used their synaesthetic experiences as material for their work, but always leaving the public short of having a first-person experience of the kind of involuntary “sensory blending” that only they seem to have enjoyed (Harrison, 2001). But could training succeed where art has so far failed?

Synaesthesia is mostly known as a relatively rare developmental condition, present in approximately 5% of the adult population. The condition appears to have a genetic basis and runs in families (e.g., Asher et al., 2009; Brang and Ramachandran, 2011). For synaesthetes, the presentation of a stimulus in a certain modality, or rather its recognition, elicits an additional atypical sensory concurrent in another unstimulated sensory channel (Cytowic, 1989/2002; Baron-Cohen and Harrison, 1997; Day, 2005; Cytowic and Eagleman, 2009). Synaesthetes have been shown to exhibit significantly different performance, as a group, in a series of behavioral tests, such as the speeded congruency test presented by Eagleman et al. (2007), designed to capture the involuntary and robust elicitation of conscious concurrents by specific inducers. The repertoire of inducer-concurrent pairings—for instance, the precise shade of color elicited by different musical notes—is usually not determined before an individual reaches the age of 9 years of age, after which time it appears to remain surprisingly consistent over the rest of the individual's lifetime (e.g., Simner et al., 2009; Niccolai et al., 2012a). Some individuals claim to have lost their synaesthesia after their teenage years, but, of all the adult synaesthetes who have been tested in contemporary research, most remember having it since childhood. Recently though, researchers have started to talk more frequently about the possibility of “acquiring” synaesthesia later in life (Ward, 2007), following short-term training, but also as a result of hypnosis, drug-use, or the extended use of certain sensory-substitution devices that systematically convert visual images into patterns of sound designed for the blind (see also Auvray and Farina, in press for discussion). As the innate character of synaesthesia is itself controversial (see Deroy and Spence, 2013a), the label “acquired synaesthesia” is potentially misleading: After all, all synaesthesia might be acquired. This is why here we consider that the real question is that of *artificially* induced synaesthesia—a label which maintains the contrast with the spontaneous forms of synaesthesia that have been documented to develop in children, and encompass cases of training, including following the use of certain conversion devices, as well as drugs or hypnosis. Possible artificially induced synaesthesia, in turn, can be distinguished from what might be better called late emerging synaesthesia that is supposed to occur spontaneously after certain kinds of brain damage or sensory deprivation (e.g., Lessel and Cohen, 1979; Jacobs et al., 1981; Bender et al., 1982; Vike et al., 1984; Rizzo and Eslinger, 1989; Harrison and Baron-Cohen, 1996; Armel and Ramachandran, 1999; Ro et al., 2007; see also Ward, 2007; Afra et al., 2009). The current article will focus on the possibility of an artificial induction of synaesthesia through associative learning. As associative learning has sometimes been complemented by drugs, or substituted for sessions of hypnotic suggestion, we will also consider the role of hypnosis and drugs, but only as far as they relate to training (see Auvray and Farina, in press; Terhune et al., in press, for broader considerations on these points). We are faced here with an alternative: have training, hypnosis and drugs been capable of artificially inducing synaesthesia or is it a mere ungrounded hope—an artificial paradise, as mentioned in the title, in reference to the famous essay where Baudelaire describes the extraordinary blending of sensory experiences he and his friends had when under the influence of drugs? (see also Gautier, 1843/1962).

Testing for the possibility of inducing synaesthesia in non-synaesthetes through training is not an end in-and-of-itself. The majority of the studies that have reached for the “acquired synaesthesia” label, either as an artificially induced form of the condition, or as a result of its emergence later in life have seemingly intended to shed some light on the processes by which a few adults end-up as synaesthetes. Given the questions that still surround the neurological (e.g., whether it comes from “*extra wires or altered function*,” Bargary and Mitchell, 2008, p. 335) and developmental origins of synaesthesia (whether it is innate or due to over-learned associations, see Baron-Cohen, 1996; Maurer and Mondloch, 2005; Cohen Kadosh et al., 2009; Holcombe et al., 2009; Deroy and Spence, 2013a), and given the higher and higher prevalence granted to the phenomenon (up to 1 on 10 once spatial and non-sensory cases are included, see Sagiv and Ward, 2006; see also Simner et al., 2006), this is a legitimate and important scientific query. However, we want to question whether the evidence collected in any of the artificially induced cases actually sheds light on the fundamental issues raised by the occurrence of synaesthesia in adults.

In the next section, we critically evaluate the problem with the series of recent studies (see Table 1 for an overview) which, despite the negative results obtained, remain ambivalent regarding the relation between these results and synaesthesia, and continue to believe that more, or better, training could perhaps one day bridge the gap between synaesthetes and non-synaesthetes. Most of them have, either explicitly or implicitly, suggested that behavioral results were sufficient to subsume artificially induced cases within the category of synaesthesia—be it as “synaesthesia” (Gebuis et al., 2009a), “pseudo-synaesthesia” (Howells, 1944; Verhagen and Engelen, 2006; Colizoli et al., 2013a), or “implicit synaesthesia” (e.g., Knoch et al., 2005). We would like to question the relevance of attaching the label “synaesthesia,” in any of its guises, to the results that have been obtained to date here. On the contrary, we argue that, if anything, it is the failure to induce a conscious synaesthetic concurrent which makes these studies interesting, as these examples help to build a contrast with synaesthesia. Indeed, what this failure suggests is that conscious concurrents come from a very rare predisposition and will not be found in the general population, and/or that there is a critical period (e.g., Daw, 2003) during which associative learning can result in the involuntary elicitation of conscious synaesthetic concurrents. We then move to a critical evaluation of a number of other ways in which, over the years, researchers have claimed that synaesthesia can be trained, namely, as a result of hypnotic suggestion or by introducing drugs during training. Here, although the occurrence of conscious concurrents is better evidenced, it is important to remember not to assimilate any apparently arbitrarily-induced conscious experiences to synaesthesia—if the latter is supposed to be distinct from involuntary crossmodal mental imagery and/or hallucinations (Spence and Deroy, 2013a).

**Table 1:** **Summary of studies that have tried to induce an association between sensory features or dimensions comparable to synaesthesia**.

**Study**	***N***	**Association (IM: intramodal; CM: crossmodal)**	**Number of trial (over what time period)**	**Behavioral/neural effect**	**Conscious concurrent**
Kelly, 1934	18	Tone-color (CM)	320–3000 (7 weeks)	No effect	No
Howells, 1944	8	Tone-color (CM)	25,000 (12 h for first 5000)	Stroop-type	In the odd participant
Nunn et al., 2002	12	Auditory words-colors (CM)	(<1 day)	No change in V4/V8 neural activity	No
Elias et al., 2003	1	Digit-color (IM)	(8 years)	Synaesthetic Stroop effect	No
Ernst, 2007	12	Visual luminance-haptic stiffness (CM)	500 (1 h)	Enhanced multisensory integration	Not assessed
Cohen Kadosh et al., 2009	4	Digit-color (IM)	Brief hypnotic suggestion	Impaired digit detection task performance	Yes
Meier and Rothen, 2009	20	Grapheme-color (IM)	10 min/day—3300 trials (7 days)	Synaesthetic Stroop effect	No
Rothen et al., 2011	20	Grapheme-color (IM)	Non adaptive, 480 trials for 10 days	Priming task (stronger after adaptive training)	No
	20	Grapheme-color (IM)	Adpative, 248 trials for 10 days		
Kusnir and Thut, 2012	28	Grapheme-color (IM)	1620 trials	Synaesthetic Stroop effect	No
Colizoli et al., 2013a	15	Grapheme-color (IM)	45 min (× 2–3 days) read, 49,000 word book (2–4 weeks)	Synaesthetic Stroop effect	Weak support
Rothen et al., 2013	1	Swimming style (pictures)-color (IM)	9600 trials (20 days)	Synaesthetic Stroop effect (no psychophysiological conditioning)	No

N = Number of participants taking part in study.

In conclusion, we stress that the inflation of new kinds of “synaesthetic” phenomena witnessed in the last decade is most probably not the right way in which to establish the scientific importance of the condition. On the contrary, we believe that this ambivalent extension potentially distracts researchers from singling out what is supposedly special about the developmental condition initially and centrally targeted by the term.

## Eight decades of failure

### Recent findings

Several recent behavioral studies have attempted to induce synaesthesia (or, as we shall see later, “synaesthesia-like behavior”) in non-synaesthetic participants as a result of training. It is worth describing these studies in some detail here.

In the first of the recent spate of acquisition studies, Meier and Rothen (2009) repeatedly exposed 20 non-synaesthetic participants to four letters (A, B, C, and D) each presented in one of four distinctive colors (red, green, yellow, and blue, respectively). The participants were presented with a total of 480 letters during training: In half of these presentations, the letters were presented in the “associated” color, while, in the remainder of the trials, they appeared in one of the three other colors. The participants had to make a speeded YES/NO discrimination response to indicate whether the color of each letter was appropriate or not. This training took place for 10 min a day over 7 consecutive days. On the eighth day, Meier and Rothen were able to demonstrate what they called a “synaesthetic Stroop effect”—that is, a significant difference in the reaction time (RT) and accuracy of participants' responses when trying to identify the color of a letter presented in a manner that was either congruent or incongruent with the intramodal associations that they had been exposed to over the course of the preceding week. Interestingly, no evidence of a conditioned synaesthetic response was demonstrated. This was measured in a synaesthetic conditioning test in which a grapheme that had been reliably associated with a particular color during training was tested to see whether or not it would, by itself, elicit a significant galvanic skin response (GSR) following a period of conditioning in which a color patch (of that particular color) had repeatedly been associated with the presentation of a loud and startling sound. By contrast, a group of thirteen grapheme-color synaesthetes exposed to a very similar training regimen in an earlier study did indeed exhibit a robust conditioning response (Meier and Rothen, 2007).

In a second study, Kusnir and Thut (2012) attempted to teach their participants a number of specific letter-color associations under somewhat less contrived experimental conditions: Specifically, the participants were not instructed about the goal of the study; What is more, the training phase of Kusnir and Thut's experiment consisted of participants performing a visual search task in which they had to make speeded left/right spatial discrimination responses to indicate the side of the screen on which a particular target letter had been presented. There were seven possible letters in total and three were presented symmetrically on either side of the screen on each trial. Unbeknownst to the participants, however, certain of the letters appeared somewhat more frequently in a given color than in any of the others^1^. Evidence that the participants had learned (or internalized) the letter-color associations that they had been exposed to during the training phase of the experiment was provided by the demonstration that color-letter congruency once again affected participants' performance in a “synaesthetic Stroop task” similar to that used by Meier and Rothen (2009). Interestingly, Kusnir and Thut (2012) went on to report interference between the learned (associated) color and the real color in which the letter was actually presented that depended upon their relative positions in color space (i.e., whether they constituted opponent vs. non-opponent colors). According to the authors, this particular aspect of their results suggested that the automatic formation of grapheme-color associations had taken place on a perceptual rather than merely on a conceptual level (Kusnir and Thut, 2012). However, it should be remembered here that opponent colors might also be more easily conceptualized as distinct, and thus, help to build two poles to which to refer the curved vs. angular letters that were the target of the study (see, for instance, the discussion in Spector and Maurer, 2011; Simner, 2012; see also Proctor and Cho, 2006).

Finally, the participants in the most recent acquisition study, this time conducted by Colizoli et al. (2013a), had to read a book (or, in a few enthusiastic cases, chose to read up to five books) in which the letters had been colored consistently (A, E, S, T in either red, orange, green, and blue; high frequency letters paired with high-frequency colors). On average, the participants read nearly 500,000 colored characters over a 2–4 week period. The specific color-letter matches used in this experiment were based on each participant's stated preference, as established at the start of the study. After having read at least one book, the participants once again exhibited behavioral congruency effects in a “synaesthetic” version of the Stroop task. They also failed to exhibit any synaesthetic release from crowding in another visual task, arguing against the existence of any low-level visual effects (cf. Cavanagh, 2001; Vatakis and Spence, 2006; Tyler and Likova, 2007).

Colizoli et al. (2013a) argued that they succeeded in inducing genuinely synaesthetic associations based on the replies given by participants to two of the 19 questions that they were asked relating to their experience of reading in color. However, it is important to note that participants' mean response to one of the questions [“*I am experiencing color when I see certain letters (in addition to the color of the text*)”] that perhaps comes closest to tapping the existence of a sensory concurrent elicited responses near the bottom of the response scale (*M* = 1.5), while their responses to the other (“*I am experiencing color when I think about certain letters*”) fell close to the midpoint of the 5-point Likert scale (*M* = 2.5). What's more, in the absence of answers to the very same questions prior to training (cf. Finke and Schmidt, 1977; Broerse and Crassini, 1984), or measures of any individual differences in the vividness of visual imagery (e.g., Marks, 1973; Craver-Lemley and Reeves, 2013; Lacey and Lawson, 2013), it is difficult to know how much these responses were actually a result of the training regimen that the participants had been exposed to, vs. a baseline tendency in certain of the participants to imagine vividly.

### Older studies

Given the long history of research on the topic of synaesthesia (see Galton, 1880, for what may well be the first popular scientific study on the condition), it is perhaps inevitable, that older references are, on occasion, simply forgotten. However, it is more than a little unfortunate that not one of the just-mentioned acquisition studies made anything more than merely a passing reference (and that only in Meier and Rothen, 2009) to the much older literature on the acquisition of “(pseudo)synaesthesia” as a result of conditioning or associative learning where more training (than in these recent studies) was, on occasion, given. Indeed, a number of researchers attempted to induce synaesthesia in non-synaesthetes back in the 1930's and 40's: Here, we are thinking particularly of the work of Kelly (1934) and Howells (1944). These early researchers attempted to establish (cross-sensory) synaesthesia, described by Howells (1944, p. 316) as a “tendency of auditory stimuli to arouse simultaneous sensations or images of color as well as of sound” in participants using a methodology that is conceptually similar to that used in the more recent studies.

So, for instance, one group of participants in Kelly's (1934) study was repeatedly exposed to a consistent mapping between each of the 8 notes constituting the C-major diatonic scale with a different color (C—White; D—Red; E—Orange; F—Yellow; G—Green; A—Blue; B—Violet; C1—White). The majority of participants were then exposed to 320 repetitions of these stimulus pairs over a period of 7 weeks. The participants were subsequently presented with the notes and asked about any spontaneous colors that they saw, and which color matched each of the notes. Kelly (1934) argued that he had not been able to induce synaesthesia (what he called artificial chromaesthesia) because of the lack of stable conscious sensory concurrents on the part of his participants—although some of those taking the mescal reported having other kinds of conscious experiences. As he put it: “Without question, the results turned out negative. They are so distinctly so that the writer has no hesitancy in concluding that it is impossible to produce chromoaesthesia in normally non-synaesthetic adult subjects by the technique of conditioned response” (Kelly, 1934, p. 336).

Now Kelly (1934) cannot be faulted for not trying here: There can't be many published studies in the field of experimental psychology that include both the unexpected firing of a pistol (blank) from close to the participant during the course of the experiment (in order to see whether putting them into a nervous state, by giving the participant “bad fright,” would increase the likelihood of synaesthesia) and the use of peyote(Lophophora williamsii, a known hallucinogen) to try to induce the condition, albeit temporarily! Now while the firing of the pistol cartridge may well have given rise to “a most admirable emotional disturbance” (p. 332), and the peyote to “gorgeous arrays of colored visions” (p. 335), not to mention severe nausea, there was absolutely no evidence of any stable conscious sensory concurrents being elicited by the presentation of specific sounds. Kelly's (1934, p. 334) conclusion on this point was unequivocal: “Not one of the eighteen subjects acquired any tendency to see colors upon the presentation of the auditory stimuli.”

A decade later, Howells (1944) pointed to the problems associated with the use of introspective report in Kelly's (1934) original study (something that Kelly himself happily admitted to). Howells, for his part, presented his participants with up to 25,000 trials of an arbitrary mapping between color and the pitch of a sound at around the same time. The participants had to discriminate a stimulus patch as being either one of two complementary colors (red vs. green) under conditions where the saturation of the colors could be varied in order to make the participants' task either easier or harder. Performance in this task was facilitated by the presentation of a sound paired with each visual stimulus that onset just before the color patch and continued during its presentation (261 Hz middle C accompanied red, and higher tone 392 Hz, the G above with green stimulus).

To minimize the risk of response bias (i.e., to avoid the possibility of participants simply responding to the tone rather than to the light) a few trials were introduced into the experimental design whereby the tone that was presented was incorrect (thus, putatively inducing errors in participants' performance in relation to the strength of the crossmodal association that had been formed). After all that training, it should come as little surprise that participants responded more slowly, and made additional errors, on those trials in which the sound playing in the background was incongruent with the crossmodal association that had been trained. In order to try to tap into any more perceptual (as opposed to response-related) effects, Howells (1944) subsequently had a couple of his participants adjust a color patch to a neutral point between red and green, or else adjust it to get the best possible white. Under such conditions, a noticeable influence of the tone playing in the background was observed, with the color being adjusted by the participants as if to make up for any color attributable to the presentation of the sound.

Here, in contrast to Kelly's (1934) earlier study, we find subjective reports suggestive of what Howells (1944, p. 101) tentatively calls pseudo-synaesthesia: “The same S also volunteered that he saw a clear image of the screen of the chromatoscope, and of the color normally associated with a given tone, when this tone was sounded while the eyes were closed or in the darkened room.” That said, the author also goes on to question “Whether or not the development of this experiment are representative of true synaesthesia, or merely what one writer has called pseudo-synaesthesia.” Indeed, what is clear from a careful reading of Howell's original study is that there was no evidence of systematic rich sensory concurrents having been consistently induced across all (or even a majority) of his participants.

The question that one might want to ask at this point is why it is that these older studies are seemingly ignored by contemporary researchers interested in inducing synaesthesia, and why it is that prophecies concerning the acquisition of genuine synaesthesia (where the presentation of a given inducer reliably gives rise to a conscious sensory concurrent) being just round the corner are still being expressed, when negative results also show up nowadays? There are certainly grounds for taking colored-graphemes as a test case for the acquisition of novel (intramodal) correspondences between sensory and/or conceptual attributes. Furthermore, it would seem logical to take the association that is by far the most common in the case of synaesthesia, present in almost 70% of self-reported cases (see Day, 2005). By contrast, trying to train a perceptual association between the pitch of sounds and different colors (Kelly, 1934; Howells, 1944) might be expected to be harder to establish, given that researchers currently believe that its' occurrence is much rarer amongst developmental synaesthetes. However, note that this line of reasoning assumes that there should be a relation between the prevalence of synaesthesia in the general population and the ease with which non-synaesthetic adults trained for a relatively brief time, typically in a laboratory study, should be able to pick-up (that is, to internalize) an association between two features/dimensions of their experience. As far as we are aware, this assumption has yet to be tested empirically. Indeed, there are certainly grounds for doubting whether the vividness of the concurrent necessarily has anything to do with the strength of the inducer-concurrent mapping (see Rader and Tellegen, 1987).

Going one step further, though, it can certainly be questioned as to whether the phenomenon of synaesthesia actually has anything at all to do with the results of these recent (and older) acquisition studies. It can be argued that all that has actually been demonstrated by these various training protocols is the learning of a novel association (either explicitly or implicitly) between a relatively small number of pairs of co-occurring sensory/conceptual features.

## Why choose the synaesthetic label?

It is important to note that none of the acquisition studies just mentioned provided any strong evidence for the occurrence of a conscious sensory concurrent. Most of these studies hesitate to claim that they have induced “full-blown” synaesthesia in any of their non-synaesthetic participants. However, our concern here is that these authors' extensive use of terms such as “pseudo-synaesthesia,” “implicit synaesthesia,” “synaesthesia-like” or synaesthetic interference is likely to confuse rather than to clarify future debate in this area. The question that we wish to pose here is what added value accrues by relating these behavioral results to synaesthesia, never mind by the addition of a range of other qualifiers?

Researchers who adopt this convoluted terminology implicitly or explicitly appeal to two arguments: First, they consider that similar behavioral aspects are sufficient to characterize a phenomenon as synaesthetic, non-withstanding the occurrence of a conscious concurrent. Second, they consider that the results of their studies do not preclude the hope that conscious synaesthesia can be induced in non-synaesthetes. However, both arguments face severe objections.

### Why behavior is not sufficient

One other way in which to defend the appeal to synaesthesia, would simply be to drop the necessity of there having to be a conscious concurrent. Recent debates over the most appropriate definition of synaesthesia and the key criteria to apply to an increasing variety of kinds and cases have led many researchers in the field to become very permissive with the use of the term (see Marks, 2011; Eagleman, 2012; Simner, 2012, for similar worries). However, of all the controversies surrounding the very existence, or definition, of synaesthesia that have been documented over the last 130 years or so (see Galton, 1880, for one of the first scientific reports on the phenomenon; see also Jewanski et al., 2009), none have questioned that the fundamental characteristic of synaesthesia is the elicitation of a conscious concurrent. It is the supplementary conscious aspect which has led to the coining of the term, which justifies the use of “aesthesia” (which means *perceiving, experiencing*). Synaesthetes are those who experience additional conscious sensory or emotional concurrents, by comparison with the experience of other individuals, in response to the actual presentation (or, on occasion, the mere imagination) of particular sensory, or conceptual, inducers. For instance, Grossenbacher and Lovelace (2001, p. 36) define synaesthesia as an “involuntary concrete sensory experience”; Simner (2012, p. 2) stresses that “These sensations are explicitly experienced in that synaesthetes are consciously aware of them in daily life”; Ward (2007, p. 429) explains that “Synaesthesia is the automatic elicitation of conscious perceptual experiences by stimuli not normally associated with such experiences”; and these are only a few of the many researchers who have stressed the central importance of the conscious concurrent to the definition of synaesthesia.

When non-conscious cases, such as crossmodal matchings or associations between apparently unrelated sensory features or dimensions of experience have been discussed in relation to synaesthesia, they have typically been recognized within their own category. Martino and Marks (2001), for example, classified them as a kind of “weak synaesthesia”—whose relation to conscious (or full-blown) synaesthesia remained an open question (see also Marks, 2011; Spence, 2011; Deroy and Spence, 2013b). It is important to emphasize here, anyway, that when researchers talk about “inducing” synaesthesia they certainly do not mean merely inducing weak synaesthesia.

The possibility of non-conscious cases of synaesthesia has also been mentioned in connection with several recent published cases of implicit bidirectional synaesthesia (see Knoch et al., 2005; Cohen Kadosh and Henik, 2006; Cohen Kadosh et al., 2007; Johnson et al., 2007; Gebuis et al., 2009a,b; Weiss et al., 2009). These studies have demonstrated that certain of the synaesthetes who consciously experience colors when seeing numbers or letters can also show specific patterns of behavioral priming for—or interference with—the processing of a letter or number when a colored stimulus is presented that happens to match the synaesthetic concurrent usually experienced in response to that number or letter. Elsewhere, Rothen et al. (2010) have reported that transcranial magnetic stimulation (TMS) over parieto-occipital brain areas can eliminate the behavioral effects of implicit bidirectional synaesthesia, as it does for the conscious cases in the reverse direction (see Muggleton et al., 2007). There is therefore, good empirical evidence that certain of the neural substrates responsible for the conscious elicitation of a concurrent also underlie the unconscious effects that have now been documented in the opposite direction. That said, while these studies may well have successfully established a non-conscious correlate of conscious synaesthesia, they have certainly not demonstrated the existence of a fully non-conscious form of synaesthesia. Note also that in the very rare, and possibly unique, case of explicit bidirectional synaesthesia studied by Cohen Kadosh et al. (2007), distinct patterns of neural activation, together with different timecourses were observed for the neural activations associated with grapheme-color and color-grapheme variants—this despite the fact that similar congruency effects were reported in both directions at the behavioral level (cf. Gebuis et al., 2009a).

The occurrence of a conscious concurrent therefore, plays a key role in the diagnosis of synaesthesia, be it established by means of questionnaire data (see http://www.synaesthesia.uwaterloo.ca/ColorAssessment.htm), or through the now seemingly well-accepted battery of tests developed by Eagleman et al. (2007). In these tests, participants have, among other things, to report on their conscious experiences. The test of consistency, for instance, often remains the standard to identify someone as a synaesthete. Although the minimal duration of consistency required to be a synaesthete can be questioned (Simner, 2012), consistency over short or long periods of time (years or series of trials, 180, for instance, in Eagleman et al., 2007) is, in the first place, a way to check the occurrence of a conscious concurrent. It is usually combined with other behavioral tests, the latter designed to confirm that a positive result on the former cannot simply be explained in terms of learned associations (e.g., Calkins, 1893; Eagleman et al., 2007), such as have been evidenced to affect participants' performance in a variety of Stroop-like interference, priming, and speeded congruency tasks (see Elias et al., 2003; Hancock, 2006; Eagleman et al., 2007). The majority of training experiments did not evidence the long-term consistency of the effects, therefore, failing to pass this test. This said, it is important to remember that even passing this test (for instance, with the 6 month positive re-test conducted by Colizoli et al., 2013a) does not demonstrate that a conscious concurrent experience necessarily occurs: Positive results might, in principle, be obtained, because of an individual's memories or non-conscious associations.

A more robust test, according to Rothen et al. (2013), comes from the transfer of a conditioning response formed with the concurrent (e.g., the association between a color and a startling sound) to the inducer (e.g., the presentation of a letter). This test establishes a strong distinction between synaesthesia and trained associations, and helps to establish the genuineness of synaesthetic reports, for instance in rare forms such as swimming style synaesthesia. As shown by this physiological test, and argued elsewhere (see Deroy and Spence, 2013b; see also Marks, 2011, for discussion), the systematic presence of a conscious concurrent in synaesthetes connects to other relevant differences in terms of how the association evolves over time.

### Why synaesthesia is not the only available label

Couldn't the behavioral effects and implicit components present in the acquisition (or teaching) cases be better conceptualized, then, in terms of the notion of cross-correspondences? Crossmodal correspondences have been defined in terms of matchings between features, or dimensions of experience across distinct senses, due to weak correlations in the environment (e.g., Spence, 2011). In contrast to what is the case in synaesthesia, crossmodal correspondences typically do not give rise to a conscious sensory concurrent (except, perhaps, in the case of those individuals with especially vivid crossmodal mental imagery; see Spence and Deroy, 2013a). Crossmodal correspondences tend to be shared amongst those individuals exposed to a particular set of environmental conditions, and those, like certain audiovisual correspondences, that are linked to robust physical or phonological regularities appear to be universal (e.g., Bremner et al., 2013).

The fact that crossmodal correspondences do not seem to immediately correlate with an obvious environmental regularity has been taken to show that they reflect innate or given predispositions (Gibson, 1969; Marks, 1978; Maurer and Mondloch, 2005; see Deroy and Spence, 2013a, for discussion). However, a better understanding of the statistics of the environment and extended models of associative learning are proving capable of explaining a large number of the cases of crossmodal correspondences that have been documented to date (e.g., see Deroy et al., in press; Parise and Spence, in press). If that is the case, why not simply say that the sort of associations learned (or strengthened) in the studies of Meier and Rothen (2009); Kusnir and Thut (2012), and Colizoli et al. (2013a) demonstrate that adults can acquire intramodal (or crossmodal, if shape and color are considered as independent channels) correspondences, instead of training them in terms of the induction of synaesthesia-like phenomena? The availability of this alternative explanation questions the fact that synaesthesia is the only existing category to enlist and study the kind of phenomena documented above.

### Why learning associations differs from developing synaesthesia

Even when they acknowledge the difference between the outcomes of training and synaesthesia, the authors of the recent training studies remain ambivalent in their conclusions: While two of the groups of researchers explicitly conclude that their results do not demonstrate that synaesthesia can be acquired (Meier and Rothen, 2009; Colizoli et al., 2013a)), all three research groups remain surprisingly upbeat about the possible acquisition of synaesthesia in adulthood—stressing that perhaps the amount of training in their studies may simply have been insufficient to elicit the full-blown condition in any of their participants. Take, for example the following quote from Meier and Rothen (2009, p. 1210): “the synaesthetic Stroop test is very useful to assess the strength of a semantic association. However, it seems to fail to assess what is unique about synaesthesia, namely the experience of the synaesthetic color”; or this from Colizoli et al. (2013a, p. 6) “these results alone are not enough to conclude the presence of synesthesia, since over-learned associations produce a Stroop effect.” However, Meier and Rothen (2009, p. 1211) concluded their study by stating that: “*it will be interesting to test whether it is possible to induce a synaesthetic experience with more training*.” Later, Rothen et al. (2011) also conclude: “*Future research using more extensive adapting training must reveal the boundaries of training synaesthetic experiences*” (Rothen et al., 2011, p. 1250).

It may be instructive to contrast those recent studies claiming to have induced synaesthesia (or one of its variants), as a result of training with the growing number of studies that have started to investigate the establishment of other kinds of correspondences (of both the intramodal and crossmodal variety) between erstwhile unassociated objects or dimensions of experience. These studies remain largely absent from the discussion initiated by the authors committed to studying the possibility of artificially inducing synaesthesia. They originate in the growing interest in trying to understand the ways in which the brain continuously updates its priors in response to changes in the statistics of the environment (e.g., Wozny and Shams, 2011; Xu et al., 2012; see also Baier et al., 2006; Van Wanrooij et al., 2010; Zangenehpour and Zatorre, 2010). Ernst (2007), for example, has demonstrated that people exposed to an arbitrary crossmodal associations between the luminance of a visual stimulus and its felt stiffness, a haptically-ascertained stimulus property (manipulated by means of a force feedback device) that is not (as far as anyone is aware) correlated with luminance in the natural environment, can give rise to a change in the strength of the coupling prior.

The participants in Ernst's (2007) study were trained with pairs of multisensory (visual-haptic) stimuli where an artificial correlation had been introduced between the two unisensory stimulus dimensions: For one group of participants, the brighter the object, the stiffer it was, while this mapping was reversed for the remainder of the participants. The results highlighted a significant change in participants' discrimination performance when their responses to congruent and incongruent pairs of haptic stimuli were compared before and after training. Ernst attributed these findings to changes in the distribution of the coupling prior. Given the very different theoretical background to this study (as compared to the acquisition studies mentioned earlier), it is perhaps unsurprising that he did not ask his participants whether the presence of one stimulus gave rise to any kind of concurrent in the other modality. Hence, at one level, we cannot say for certain whether or not Ernst's participants acquired any synaesthesia-like abilities following training.

Now, clearly, the experimental paradigm utilized by Ernst (2007) exhibits some important differences from the other acquisition-by-training studies that were mentioned earlier. Namely, in contrast to the other acquisition studies, where particular stimuli were associated (i.e., without there being any alignment of dimensions of experience, except for Kelly (1934, pp. 323–324) who did try to align his auditory and visual stimuli but did so using what is known as the Newtonian parallel: “*in order to take advantage of any possible existent relationship between color and tone (generally known as the Newtonian parallel) it was decided to pair each note of the octave with the color occupying the same relative position in the spectral series as the note occupies in the musical scale.*”) Ernst, by contrast, used correlated dimensions of experience. Hence, his participants presumably learned the appropriate alignment (at a perceptual level) between two already familiar dimensions and, in so doing, likely demonstrated a generalization of learning beyond the particular set of stimuli that participants had been exposed to during training.

In other words, training studies interested in synaesthesia rely on associative learning to form a one-to-one matching between two limited sets of dimensions or objects, or what one might call a bijection: Each letter should be associated to a different color, and each color to a different letter. By contrast, studies interested in new binding priors rely on associative learning to recalibrate an existing mapping, where each and every value on one dimension, even beyond the learned ones, will correspond to at least one value on the other dimension (with the possibility of two different values on one dimension corresponding to a single value on the other, because of differences in accuracy). This contrast helps pointing out that training studies for synaesthesia are—at least—implicitly committed to synaesthetic associations being of the matching rather than the mapping form.

Now, if one adopts the view that color-grapheme synaesthesia only comes as a late specification growing out of what can be considered as a much more general mapping from degrees of angularity to degree of brightness (Spector and Maurer, 2008, 2011), this commitment might need to be revisited. Would it, for instance, be better to gradually train participants with more and more specific associations between shapes and colors, up to specific associations between graphemes and individual shades? None of the studies which have claimed to have induced some association related to synaesthesia has been particularly interested in testing this specific prediction, because they all choose to start with the synaesthetic inducers known in adults, that is, with graphemes. Notice here that synaesthetes can report having the same color for different letters, suggesting also that the one-to-one correspondence is not necessarily the right way to think about the determination of associations.

Finally, in all of the training cases, we wish to argue that what these training studies miss is the resistance of synaesthesia to further exposure: Once graphemes have been associated with a specific color, synaesthetes cannot simply be retrained to associate them to new colors, nor does their synaesthesia seem to be distorted by novel exposure. This point is particularly important as it highlights, in our sense, what might be special about the induction of a conscious concurrent in developmental synaesthetes, that is, that the association is constantly reinforced every time a grapheme is seen, and appears with the concurrent color (whereas the association will fade in non-synaesthetes, who will just see the grapheme in its actual color).

### What we win from resisting the synaesthetic label

Instead of looking for—or continuing to prophesy about—the possible occurrence of a conscious concurrent after training a particular association, the decision to qualify the effects of training as being “synaesthetic” at all should impinge on empirically testable hypotheses.

Besides the conceptual issues, the question also has methodological implications regarding the choice of stimuli and associations to be used in training studies. Noticeably, in order to decide that idiosyncratic synaesthetic concurrent-inducer relations can be quickly trained, future studies should presumably avoid using those stimuli that happen to follow existing intra- or crossmodal correspondences (which, in this case, would only be strengthened through training). One reason to raise this worry comes from Colizoli et al. (2013b) who mentioned the existence of a “synaesthetic” Stroop effect even before training. Out of the four color-letter associations tested by Meier and Rothen (2009), three correspond to the implicit associations shared by many synaesthetes (see Simner et al., 2005): A's are commonly red, C's yellow, and D's blue for non-synaesthetes as well as for synaesthetes. One way in which to see whether the strength of the training effect reported in Rothen and Meier's study might have come from the reinforcing of pre-existing correspondences in the non-synaesthetic participants would be, then, first to test participants for their letter-color associations prior to testing, and at least to compare the results for these three letters with the non-common association of B and green (B's are usually associated with blue). This would not directly solve the issue of whether a conscious concurrent can be elicited after training, but would at least help to determine whether crossmodal correspondences have something to do with the results.

A more directly testable empirical prediction to draw out here is that if a synaesthesia-like phenomenon is at stake in the training studies, rather than an intramodal correspondence, then it should show a high degree of automaticity. Contrary to what appears to be the case for synaesthesia, it is indeed questionable whether or not crossmodal correspondences are automatic, i.e., goal-independent and load-insensitive (see Spence and Deroy, 2013b, for a review; see also Mattingley, 2009). Differences in reaction-time (RT) in Stroop-like tasks or other behavioral paradigms could be used to compare the kind of “trained” relations that are labeled as “synaesthetic” and cross- or intra-modal correspondences, but this should be done with equally trained participants (some reporting visual concurrents vs. others who do not) given independent evidence that the amount of training increases the RT differences between congruent and incongruent conditions in Stroop-like tasks (MacLeod and Dunbar, 1988; Colizoli et al., 2013b).

As a second test, one might consider whether the behavioral results from these (or future) acquisition studies shouldn't be combined with neurological data: Although both synaesthesia and crossmodal correspondences have been shown to be disturbed by TMS over the appropriate brain areas, the disturbance of synaesthesia has been shown to result from the disruption of neural activity in the right parieto-occipital (PO) junction (Muggleton et al., 2007). By contrast, the disturbance of the crossmodal correspondence between pitch and brightness in Bien et al's. (2012) study was targeted at the intraparietal sulcus (IPS) instead. Crossmodal correspondences present relevant differences with synaesthesia that are now progressively being documented. Studies highlighting differences between synaesthetes and non-synaesthetic participants trained with similar associations (e.g., Nunn et al., 2002; Elias et al., 2003) again argue in favor of the non-assimilation of artificially induced cases with synaesthesia, and thus, encourage more systematic comparison between naturally-occurring crossmodal and intramodal correspondences, evidenced in non-synaesthetic populations, and the more ephemeral cases of trained pairings of formerly uncorrelated stimuli (or, as we have seen, the strengthening of weakly associated stimuli). Based on the findings of animal neurophysiology (e.g., Furster et al., 2000), one might predict that the acquisition of novel cross-modal associations between formerly independent auditory and visual features (e.g., a red light being paired with a high tone and green light with a low tone) might recruit/require the involvement of the dorsolateral prefrontal cortex. That said, different areas may be involved in the establishing vs. retention of correspondences. Once firmly established, correspondences may be “represented” at more posterior locations, such as in parietal areas (see Bien et al., 2012).

### Interim summary

In summary, despite the growing support for there being robust, most often non-conscious crossmodal correspondences in adults and implicit effects of the concurrent on responses to the inducer (especially in the case of intramodal grapheme-color synaesthesia), there is, as yet, no convincing evidence to support the claim that our definition of the condition (synaesthesia) ought to relax the necessity of a conscious concurrent. What is more, it is perhaps also worth reiterating that the recent acquisition studies that were reviewed earlier all agree that a consistent conscious concurrent is a key part of what it is to be synaesthetic. Take, for example, the following from Meier and Rothen (2009, p. 1210) in support for this view: “*we conclude that the synaesthetic Stroop test is very useful to assess the strength of a semantic association. However, it seems to fail to assess what is unique about synaesthesia, namely the experience of the synaesthetic color.*”

Next, we turn our attention to some of the other modes of training in which researchers have claimed that experiences of synaesthetic colors, and synaesthetic concurrents more broadly conceived, can be artificially induced, namely through the use of hypnotic suggestion and by complementing training with the administration of a drug.

## Hypnotic suggestion and additional drugs: new prospects for artificially induced synaesthesia?

### Hypnotically induced concurrents

An influential recent study investigated whether it is possible to induce synaesthesia by simply instructing non-synaesthetes under hypnotic suggestion to associate certain colors with particular graphemes. Cohen Kadosh et al. (2009) conducted an experiment on a small group (*N* = 4) of highly hypnotically suggestible non-synaesthetes (these participants had been screened, and had to have obtained the maximum score on the Stanford Hypnotic Susceptibility Scale prior to their taking part in the main part of the study). These participants were hypnotized by Cohen Kadosh et al., and while under hypnosis, instructed to associate specific digits with specific colors (just as done by grapheme-color synaesthetes—here 1-red; 2-yellow; 3-green; 4-turquoise; 5-blue; and 6-purple). Then, during post-hypnotic suggestion, various tests were administered, including a digit detection task in which a sequence of achromatic digits was presented against one of a number of colored backgrounds. On half of the trials, a digit was presented, and the participants had to make a YES/NO discrimination response regarding its presence vs. absence. Using such a task, the authors calculated a measure of perceptual sensitivity (*d'*) using signal detection theory: Crucially, a significant drop in participants' performance was observed when the letters were presented against a background that matched (that is, was congruent with) the color that had been associated with the digit while the participants were under hypnosis than when presented against an incongruently colored background. By contrast, no such drop in performance was reported in any of the control groups tested in this study.

Subjective (i.e., phenomenological) reports were also obtained from the participants. As Cohen Kadosh et al. (2009 p. 263) report: “*At the phenomenological level, participants' reports after the posthypnotic suggestion matched those observed in congenital synaesthetes … The cross-modal experience was consistent and involuntary, and occurred in their everyday life. For example, one participant reported seeing the digit-color associations when looking at cars' license plates or watching television.*” This despite the fact that the post-hypnotic participants didn't remember having been instructed concerning any digit-color associations. It is, however, unclear from the above quote, or from the rest of the text which aspect of consistency is being referred to here—is it that whenever a concurrent was induced it was consistent in its color and/or was some concurrent (whether or not it was the same one) always induced on seeing a given inducer?

We would argue that Cohen Kadosh et al.'s (2009) results provide what is perhaps the strongest evidence to date that non-synaesthetes can be induced to exhibit behaviors that resemble those observed in synaesthetes, given the apparent elicitation of conscious sensory concurrents. Bear in mind, though, that the authors themselves stop short of claiming that they had been able to induce synaesthesia, even in their highly suggestible participants. Instead, they merely state that: “*Here we show that posthypnotic suggestion induces abnormal cross-modal experience similar to that in congenital grapheme-color synaesthesia*.” (Cohen Kadosh et al., 2009, p. 258, see also p. 263). What is more, an alternative explanation for Cohen Kadosh et al.'s striking results exists; Namely, it is difficult to rule out the possibility that their participants were not simply engaging in a particularly vivid form of visual mental imagery rather than necessarily experiencing synaesthetic concurrents. The instructions that were given to the participants could equally well be associated with the hypnotic induction of mental imagery as with the hypnotic induction of some sort of projector synaesthesia. So, for example, take Cohen Kadosh et al.'s (2009, p. 260) instructions to their participants: “*Look at the color; this is the color of the digit _, and whenever you see, think, or imagine that digit, you will always perceive it in that color.*” Remember here that highly suggestible individuals also tend to have more vivid visual mental imagery (e.g., Crawford, 1982; Rader and Tellegen, 1987), and that visual imagery has been shown to affect visual perceptual sensitivity in a number of studies (e.g., Ishai and Sagi, 1995).

Again, something conceptually similar to Cohen Kadosh et al. (2009) had been reported previously. In particular, Leuba (1940) paired the rubbing of a participant's arm while under hypnosis with the smell of creosote. Later, the participant had an olfactory image of creosote whenever his arm was rubbed, although he too had no memory of making this crossmodal association. Interestingly, Leuba describes this as an example of conditioned mental imagery (see also Cytowic, 1989/2002, pp. 67–68).

What's more, it is not clear, from Cohen Kadosh et al.'s (2009) report whether any of the highly hypnotically suggestible participants might not also have had similar unusual phenomenology prior to their hypnotic suggestion (cf. Finke and Schmidt, 1977; Broerse and Crassini, 1984, for a very different example of where apparently experiment-induced color percepts, actually turned out to be present prior to experimental manipulation of interest, thus, perhaps acting as something of a cautionary note here). Ever since its appearance in the scientific literature, it has proven particularly difficult to distinguish between synaesthetes and those with especially vivid mental imagery (see Galton, 1880; Vernon, 1937; Craver-Lemley and Reeves, 2013; Price, in press; Spence and Deroy, 2013a). Nevertheless, despite the difficulty of drawing this distinction, many researchers clearly feel it is important to do so.

Future neuroimaging studies might be able to distinguish between these two accounts in the case of color concurrents/color imagery induced by the presentation of a given stimulus, given that color imagery seems to activate an area of the brain that lies substantially anterior to the classic V4/V8 region thought to be involved in color perception and often active in synaesthetes when experiencing conscious color concurrents (see Howard et al., 1998; Nunn et al., 2002; Rich et al., 2006; Niccolai et al., 2012b).

### Drug-induced concurrents

Drugs have recently been used as another example, and possibly a distinct route, to the induction of synaesthesia, following perhaps the long tradition in writers and artists to consider drugs, a form of sensory mixing and creativity (e.g., Dailey et al., 1997; Sitton and Pierce, 2004). Importantly, the induction of synaesthesia by drugs (often taken as a fact) has been used to constrain possible accounts of the neural underpinnings of synaesthesia (Grossenbacher, 1997; Grossenbacher and Lovelace, 2001). Here, though, we only want to consider the use of drugs in the light of the training studies (see Terhune et al., in press, for a recent review).

In Kelly's (1934) early study, peyote was used after training to see whether the predicted occurrence of hallucinatory experience while the drug was active would follow the conditioned association (trained in the absence of the drug). But, of the five participants who volunteered to take 15 g of peyote, none reported experiencing the learned color when the sounds used for training were played (although Kelly, 1934, p. 335, does say that his participants were “*rewarded with gorgeous arrays of colored visions which compared favorably with the ones described by previous investigators of the so-called divine plant*”). This failed attempt to combine training and induction of conscious concurrents contrasts with a more difficult to assess, but certainly more up to current ethical standards, survey regarding whether people might have synaesthetic experience while under the influence of certain pharmacological agents, and this independently of any preliminary short-term training with certain specific associations (see Luke et al., 2012). These researchers documented correlations between the frequency of drug consumption, the kinds of drugs consumed, and subjective reports of abnormal crossmodal experiences in recreational drug users. On the basis of their survey, Luke et al. argued that: “*synaesthesia is frequently experienced following the consumption of serotonergic agonists such as LSD and psilocybin and that these same drugs appear to augment synaesthesia in congenital synaesthetes*.” (Luke et al., 2012, p. 74). As serotonin (5-HT) is a neurotransmitter with mostly inhibitory effects, and yet some documented excitatory effects as well, it is difficult to use this correlation to decide between competing models of synaesthetic induction—in particular, between Grossenbacher and Lovelace's (2001) disinhibited feedback account and Ramachandran and Hubbard's (2001; Hubbard et al., 2011) direct cross-activation account. One important test here would be to investigate the combined effect of serotonin and training in the induction of conscious concurrents.

This said, if the use of drugs were to open new prospects for the elicitation of conscious experiences in otherwise non-synaesthetic participants, that would raise important questions regarding the kind of conscious concurrents that should be considered as synaesthetic. Sinke et al. (2012) have recently listed a number phenomenological differences between the experiences occurring in developmental synaesthesia and those reported in what they still consider as a separate type of synaesthesia, induced by drugs. The main lesson of their review, in our sense, is that the unreliability of the reports and/or the variability of drug experiences in terms of temporal dynamics and contextual or individual differences (including the quantity and quality of drugs taken) might not even authorize a systematic comparison, even within a single class of drugs. In this respect, we would recommend to stick to behaviorally measurable elements, or neurological data.

Furthermore, unless a form of consistency, through behavioral tests such as Eagleman et al. (2007) speeded congruency test, is established, the connection with synaesthesia remains terminological, and no continuity or relation between drug-induced and non-drug induced cases can be firmly established. How such tests could be reliably performed with participants under the influence of a given pharmacological agent is, however, a source of both ethical and practical concern. The alternative description for such results is that taking one of these hallucinogenic drugs leads to nothing more than vivid hallucinations (see MacDougal, 1898; Aghajanian and Marek, 1999). The evidence here could come from resting-state fMRI, which shows an increased intrinsic functional connectivity in synaesthetes similar to that which is observed in schizophrenic patients who are subject to hallucinations (Jafri et al., 2008).

### The benefits of resisting the synaesthetic label

As the occurrence of conscious concurrents remains definitional and mysterious in synaesthetes, and as synaesthesia is a naturally occurring phenomenon that is not known to correlate with drug usage or hypnotic training, it can be argued that the former studies do not really shed any light on the core of synaesthesia. Is it however, possible that the brain mechanisms at stake in the hypnotic and drug induction of conscious concurrents are similar to—or at least serve as some sort of proxy for—the mechanisms explaining the development of synaesthesia?

As far as this suggestion is concerned, it is unclear whether the results of the hypnotic and drug studies have actually shed any light on the fixation of the synaesthetic repertoire, given the lack of any documented consistency over time for the pairs of inducers and concurrents. In Cohen Kadosh et al.'s (2009) study, the effect only lasted for 3 weeks. The same is true, notice, of Colizoli et al.'s (2013a,b) more controversial results, as they reported that the associations that their participants had “learned” had disappeared within 6 months of training having terminated. This, once again, obviously contrasts with claims regarding the persistence of synaesthetic pairings in developmental synaesthesia, which usually lasts a lifetime (e.g., Ward, 2013).

Besides consistency, we should not miss some important differences between the kinds of conscious experiences obtained after hypnosis or drug consumption and the kinds of conscious concurrents reported in synaesthesia. For instance, a vast majority of synaesthetes report experiencing the concurrents “in their mind's eye” and as vividly as perceptual experience—two reports that they do not use to characterize their mental imagery. In the case of the induced concurrents experienced after drug consumption, it is unclear whether the participants were in a state where they could reliably assess the vividness of the experience, independently of the heightened emotional state also induced by the drug. No data is given regarding the peculiar location of the concurrent (external, internal, or “in the mind's eye”). In the case of the induced concurrents following hypnosis, assessment of the vividness of the experience is missing, and the concurrents seem to be experienced as external—a case of projection which is rare or even controversial (Hupé et al., 2012) in the case of synaesthesia. Another difference between the phenomenology of mental imagery and the most common features of developmental synaesthetic cases, is to see whether the experienced form can be zoomed in upon (a signature feature of spatial mental imagery; Finke and Shepard, 1986), whereas synaesthetic concurrents can usually not be changed volitionally (see Price, 2009, for a similar suggestion).

What this suggests is the need to distinguish between the elicitation of certain forms of mental imagery, including crossmodal imagery and synaesthesia. Crossmodal mental imagery is defined as mental imagery occurring in one sensory modality as the result of the presentation of a stimulus in another sensory modality. The occurrence of auditory mental imagery when watching silent speech, or the occurrence of visual imagery when walking in the dark e.g., Sathian and Zangaladze, 2001; Zhang et al., 2004; for neuroimaging evidence, see Lacey et al., 2010; Lacey and Sathian, 2013, provide good examples of crossmodal imagery. It often serves a role of crossmodal completion, by filling-in the missing features of a stimulus that is physically present (e.g., Pessoa and De Weerd, 2003; Gallace and Spence, 2011; see Spence and Deroy, 2013a, for a review) but it can also occur in less specific ways as shown in the elicitation of visual or proprioceptive imagery in musical experience.

Here, we want to side with Price (2009) in suggesting that the effects of developmental synaesthesia should be kept distinct from the mere effects of mental imagery. Although Galton (1880) became interested in colored-hearing as a case of mental imagery, most definitions since have considered synaesthesia to be something different from highly consistent, highly automatic mental imagery. Even Barnett and Newell (2008) who document higher self-rated visual imagery in color-grapheme synaesthetes stress that synaesthesia should be kept distinct from mental imagery. The underlying mechanisms of synaesthesia are distinct from the ones involved in classical mental imagery tasks (see Nunn et al., 2002; Rich et al., 2006; Hupé et al., 2012) and the resulting concurrent usually considered as perceptual, rather than imagistic (Auvray and Deroy, in press for a review).

We recommend, first, that results of hypnosis and reports from past drug experiences—as well as future training studies—should be analyzed with respect to participants' mental imagery scores. Taking number-space as an example, there is growing evidence that some—or all—individuals classified as synaesthetes might just have a particularly vivid and consistent form of spatial or visual imagery, which can be tested independently (Price, 2009). Participants identified with a possible form of synaesthesia, should also be tested behaviorally (for instance, using Stroop-tests) while being instructed to imagine a congruent vs. incongruent color (or concurrent). In so doing, Price, for instance, has shown that many participants identified as “number-space” synaesthetes because of their spontaneous subjective reports of experiencing numbers in space would perform in a way that is similar to other participants with high visual mental imagery in congruent and incongruent imagery instructions. Price goes on to suggest that instead of synaesthesia, odd case reports of seeing numbers in space could be explained in terms of “*the interaction between (1) a predisposition for exaggerated and sometimes distorted spatial coding of numerical or temporal sequences, and (2) the strong visual imagery needed to make these representations salient and explicit*” (Price, 2009, p. 1239).

## Conclusions

In conclusion, in this article, we have questioned the utility (not to mention the validity) of talking about learned associations between erstwhile independent sensory features (or dimensions of experience) in terms of “pseudo-synaesthesia,” “synaesthesia-like,” or “acquired synaesthesia” that has become increasingly popular over the last few years (e.g., see Meier and Rothen, 2009; Kusnir and Thut, 2012; Colizoli et al., 2013a). It is our position that one can avoid the terminological confusion [not to mention the implications of adding to the problematic unity (see Marks, 2011), and etiology of synaesthesia (see Maurer and Mondloch, 2005; Deroy and Spence, 2013a)]. The effects of training can be more easily accounted for within the category of crossmodal and intramodal correspondences, and explained in terms of coupling priors in Bayesian Decision Theory (e.g., Ernst, 2007).

The expansion of different qualifiers for, or divisions between kinds of, synaesthetes over the last decade or so has tended to blur the definition of the condition—while most of these divisions remain controversial, as shown for “associator” and “projector” synaesthetes (Dixon et al., 2004; Rouw and Scholte, 2007; Ward et al., 2007; though see also Gebuis et al., 2009a), “lower” and “higher” synaesthetes (Ramachandran and Hubbard, 2001; Hubbard et al., 2005; Gebuis et al., 2009a), “explicit” and “implicit” synaesthetes (Knoch et al., 2005), and “weak” and “strong” synaesthetes (Martino and Marks, 2001; but see Deroy and Spence, 2013b). Ultimately, despite many researchers having written over the years as if artificially induced synaesthesia is a well-established condition, as compared to the much more common (albeit still rare) developmental (sometimes called congenital or idiopathic synaesthesia) variety, it is possible to raise legitimate grounds for pursuing the argument that there may actually be no such thing as “becoming a synaesthete” in neurotypical adults (see also the evidence for a genetic basis of synaesthesia, Asher et al., 2006, and distinctive neurological profile, Rouw and Scholte, 2010). In other words, it may be impossible to acquire synaesthesia unless one is *predisposed* to be a synaesthete and hence develops it during a critical period of development.

A claim of impossibility seems difficult to support empirically. After all, three possibilities exist here: (1) It is impossible to acquire synaesthesia in adulthood, at least if synaesthesia is defined with respect to the criteria established on the basis of studies of developmental synaesthesia (and hence robust supporting evidence will simply never be forthcoming); (2) It is possible to acquire synaesthesia in adulthood, but it is just that robust empirical evidence has not as yet been established to support this claim; (3) It is evident that non-synaesthetes can become synaesthetes, and we simply need to change our definition in order to take account of the kinds of expressions of the condition that are seen in acquired cases. This third option is the one which seems to encourage the premature labeling of the effects of training as synaesthetic. In the absence of robust evidence of a successful artificial induction of synaesthesia, the idea behind the premature label is that training studies are nonetheless already teaching us something about synaesthesia—for instance, that Stroop interference between apparently unrelated features is not a defining characteristic of synaesthesia. This seems to us a weak argument given the long literature on training and Stroop interference written independently of any concern for synaesthesia. On the contrary, these training studies highlight how different synaesthetic pairings are from learned associations, noticeably that they resist further learning through exposure (Deroy and Spence, 2013a) and can lead to associatied conditioned responses (Meier and Rothen, 2009).

The first claim, that training will never bridge the gap with synaesthesia, is certainly a controversial one, but the one that the accumulated evidence seems to favor. What about the possibility (2) that future hypnosis, or carefully-controlled drug studies might provide evidence of consistent, idiosyncratic, automatic, and crucially conscious concurrents being induced by specific sensory (or conceptual) inducers? As we have stressed in this piece, the evidence in support of such a claim is, at present, remarkably thin on the ground. Regarding the most promising example to date of acquired synaesthesia (Cohen Kadosh et al., 2009), it remains to be seen whether this is anything more than merely hypnotically-induced mental imagery (cf. Leuba, 1940), though the distinction here is, admittedly difficult to draw out (Craver-Lemley and Reeves, 2013). What would nonetheless be the consequences if robust cases of artificially induced synaesthesia were to be evidenced? One possible consequence would be to consider that the idea of a neurological structural grounding for synaesthesia (e.g., Banissy et al., 2012) does not hold, as individuals without the neurological (qua genetic) disposition to develop synaesthesia in childhood could acquire it later in life. The evidence of a polygenetic origin for synaesthesia—still weak (Brang and Ramachandran, 2011)—could point toward a second possibility, that the disposition to become a synaesthete is not equally distributed in the population and in time.

For the moment though, the inclusion of the results of training, drugs, or hypnosis adds to the fact that the word synaesthesia is acting as something of an umbrella term, and losing its scientific robustness (see Table 2). The way to resist this dissolution is to insist on the occurrence of a conscious concurrent being a necessary condition for meriting the label of synaesthesia. A form of consistency should be present, not as an *a priori* definitional demand (which can be seen as a dogmatic threshold, see Simner, 2012), but as a way to give weight to the occurrence of the conscious concurrent (see Eagleman et al., 2007).

**Table 2:** **Illustration of the various kinds of phenomena that have been labeled as synaesthetic in the literature—and how they depart from the characteristics of developmental/canonical synaesthesia**.

**Name used in the literature**	**Origin**	**Conscious concurrent**	**Consistent over long periods of time**	**Idiosyncratic**	**Involuntary**	**Rare**
Canonical synaesthesia	Developmental	Yes	Yes	Yes	Yes	Yes
Acquired synaesthesia	Training	No	No	n.a.	Yes	n.a.
Acquired synaesthesia	After brain damage or sensory deprivation	Yes	Varying Yes	Yes	Yes	Yes
Acquired synaesthesia	Extensive use of a sensory substitution device	Yes	Weak evidence	No	Weak evidence	Yes
Acquired synaesthesia	Drug	Sometimes	No	Yes	Yes	Unknown
Weak synaesthesia	Debated	No	Yes	No	Debated	No
Neo-natal synaesthesia	Innate	Weak evidence	Yes	No	Yes	No

This table shows that, even in the broad category of “acquired synaesthesia,” what we suggest calling “artificially induced synaesthesia” presents more differences with canonical synaesthesia than other novel conditions sometimes labeled as emergent forms of synaesthesia.

Now, if it is impossible to acquire synaesthesia in adulthood, how can we explain the fact that many if not all of the inducer-concurrent mappings exhibited by so-called developmental synaesthetes must have been acquired at some stage during development? Think only of the acquisition of colored graphemes, or the childhood tastes (rather than, say, the tastes associated with baby food), that can act as inducers/or concurrents in certain synaesthetes. Here perhaps the appropriate thing to do is to suggest that there may simply be a critical period in human development for the acquisition, or expression, of developmental synaesthesia (see Daw, 2003, on the notion of critical periods for visual development)—probably only in those with a genetic disposition to develop it (Brang and Ramachandran, 2011). There is only one documented case of discordant monozygotic female twins, where one sister has conscious grapheme-color synesthesia while the other does not (Smilek et al., 2005) But this is not a counterexample showing that individuals could have the genetic disposition and not develop synaesthesia: As the authors themselves point out, the absence of development must also have a genetic (and not environmental) basis (i.e., an epigenetic event, X chromosome inactivation, or a mutation of a synaesthesia gene).

For us, perhaps the more interesting issues in this area revolve around the question of if, and why, it may be easier to acquire certain associations than it is to acquire others, and whether there is any relation between the associations that appear commonly in developmental synaesthetes and those that non-synaesthetes find it easier to acquire (or else to strengthen). One other question that seems particularly interesting in terms of future study relates to individual differences in the ability of people to learn new associations (be they intramodal or crossmodal). This issue has come up in passing in a number of the studies that have been reviewed here (e.g., Kusnir and Thut, 2012; see also Cohen Kadosh et al., 2009). It might well turn out to be the case that certain associations are just easier to learn that others (cf. Baeyens et al., 1990)—such as, for example, the association between rounded shapes or higher pitch sounds and brighter colors vs. angular shapes and lower pitch sounds and darker colors. This would then explain why these higher-order are resemblances found between synaesthetes, and between synaesthetes and non-synaesthetes (Simner et al., 2005).

As far as the explanation of the development of synaesthesia is concerned, we would argue the real challenge is to understand the specificity of the learning curve of synaesthesia (including its resistance to further training and its high degree of stability over time once established) as well as the specific nature of the concurrent experience.

### Conflict of interest statement

The authors declare that the research was conducted in the absence of any commercial or financial relationships that could be construed as a potential conflict of interest.

## Footnotes

^1^In fact, the pairings between the letters “H” and “U” and the colours blue and red were not systematic but rather just statistically more frequent, and inserted among other colour-letter pairings that were varied randomly (i.e., the letters were uncorrelated with the colour in which they happened to be presented) on a trial-by-trial basis. Note also that the pairings varied from one participant to the next (H-blue or red, U-blue or red).
